# Supplementary material for: 1H-NMR Based Serum Metabolomics Identifies Different Profile between Sarcopenia and Cancer Cachexia in Ageing Walker 256 Tumour-Bearing Rats
Source: Metabolites. 2020 Apr 21;10(4):161. doi: 10.3390/metabo10040161 (PMC7240940; doi:10.3390/metabo10040161)
Supplement: Supplementary file 1 [file metabolites-10-00161-s001.pdf]

Table S1. Total serum metabolic profile identified in adult, ageing and ageing Walker 256 tumour-bearing rats.

**Metabolite**	**Accession number**	**A**	**S**	**Wi**	**Wa**	***P*-value**
		**Mean ± SD (mM)**	**Mean ± SD (mM)**	**Mean ± SD (mM)**	**Mean ± SD (mM)**	
2-Hydroxybutyrate	HMDB00008	0.002 ± 0.001	0.002 ± 0.001	0.008 ± 0.009	0.015 ± 0.019	0.263
2-Hydroxyisovalerate	HMDB00407	0.002 ± 0.0001	0.001 ± 0.001	0.003 ± 0.003	0.011 ± 0.015	0.265
2-Oxoglutarate	HMDB00208	0.010 ± 0.003	0.016 ± 0.005	0.007 ± 0.004	0.006 ± 0.002	**<0.001**
3-Hydroxybutyrate	HMDB00357	0.022 ± 0.016	0.062 ± 0.025	0.127 ± 0.176	0.188 ± 0.299	0.554
3-Hydroxyisobutyrate	HMDB00023	0.004 ± 0.002	0.010 ± 0.003	0.009 ± 0.005	0.012 ± 0.008	0.155
Acetate	HMDB00042	0.148 ± 0.220	0.021 ± 0.011	0.037 ± 0.020	0.032 ± 0.015	0.088
Acetoacetate	HMDB00060	0.005 ± 0.001	0.012 ± 0.010	0.043 ± 0.094	0.120 ± 0.209	0.441
Acetone	HMDB01659	0.001 ± 0.0004	0.006 ± 0.001	0.019 ± 0.036	0.050 ± 0.085	0.383
Alanine	HMDB00161	0.090 ± 0.049	0.245 ± 0.047	0.182 ± 0.037	0.151 ± 0.029	**<0.001**
Allantoin	HMDB00462	0.027 ± 0.010	0.038 ± 0.003	0.124 ± 0.209	0.139 ± 0.232	0.672
Arginine	HMDB00517	0.030 ± 0.020	0.052 ± 0.009	0.046 ± 0.018	0.041 ± 0.016	0.254
Asparagine	HMDB00168	0.016 ± 0.004	0.041 ± 0.007	0.020 ± 0.008	0.017 ± 0.004	**<0.001**
Aspartate	HMDB00191	0.009 ± 0.005	0.019 ± 0.003	0.013 ± 0.003	0.010 ± 0.003	**<0.001**
Betaine	HMDB00043	0.035 ± 0.020	0.112 ± 0.027	0.066 ± 0.024	0.072 ± 0.036	**0.009**
Carnitine	HMDB00062	0.010 ± 0.006	0.027 ± 0.006	0.018 ± 0.004	0.016 ± 0.005	**0.001**
Choline	HMDB00097	0.005 ± 0.003	0.012 ± 0.004	0.008 ± 0.003	0.007 ± 0.003	**0.021**
Citrate	HMDB00094	0.038 ± 0.020	0.147 ± 0.020	0.097 ± 0.036	0.080 ± 0.029	**<0.001**
Creatine	HMDB00064	0.056 ± 0.030	0.107 ± 0.051	0.115 ± 0.145	0.124 ± 0.144	0.829
Creatinine	HMDB00562	0.005 ± 0.001	0.016 ± 0.002	0.020 ± 0.022	0.015 ± 0.011	0.554
Dimethylamine	HMDB00087	0.027 ± 0.008	0.001 ± 0.0001	0.002 ± 0.002	0.002 ± 0.002	**<0.001**
Ethanol	HMDB00108	0.137 ± 0.081	0.111 ± 0.040	0.129 ± 0.033	0.148 ± 0.068	0.701
Formate	HMDB00142	0.099 ± 0.167	0.026 ± 0.007	0.023 ± 0.008	0.027 ± 0.011	0.179
Fumarate	HMDB00134	0.002 ± 0.002	0.003 ± 0.002	0.001 ± 0.001	0.001 ± 0.0005	**0.039**
Glucose	HMDB00122	0.881 ± 0.478	1.763 ± 0.537	1.725 ± 0.634	1.361 ± 0.731	0.141
Glutamate	HMDB00148	0.036 ± 0.022	0.087 ± 0.023	0.056 ± 0.025	0.037 ± 0.010	**0.001**
Glutamine	HMDB00641	0.107 ± 0.061	0.374 ± 0.065	0.238 ± 0.066	0.192 ± 0.076	**<0.001**
Glycerol	HMDB00131	0.109 ± 0.054	0.458 ± 0.061	0.244 ± 0.073	0.303 ± 0.131	**<0.001**
Glycine	HMDB00123	0.052 ± 0.027	0.141 ± 0.027	0.086 ± 0.017	0.095 ± 0.032	**0.001**

Histidine	HMDB00177	0.019 ± 0.004	0.030 ± 0.018	0.024 ± 0.010	0.033 ± 0.023	0.545
Isoleucine	HMDB00172	0.018 ± 0.008	0.041 ± 0.006	0.030 ± 0.007	0.024 ± 0.007	**<0.001**
Lactate	HMDB00190	1.720 ± 1.541	5.588 ± 1.510	3.192 ± 0.856	2.404 ± 0.499	**<0.001**
Leucine	HMDB00687	0.027 ± 0.013	0.063 ± 0.007	0.047 ± 0.011	0.051 ± 0.017	**0.007**
Lysine	HMDB00182	0.088 ± 0.056	0.163 ± 0.027	0.117 ± 0.027	0.093 ± 0.018	**0.003**
Methionine	HMDB00696	0.014 ± 0.007	0.030 ± 0.004	0.021 ± 0.006	0.018 ± 0.002	**<0.001**
myo-Inositol	HMDB00211	0.026 ± 0.010	0.055 ± 0.015	0.045 ± 0.037	0.072 ± 0.086	0.613
N,N-Dimethylglycine	HMDB00092	0.002 ± 0.001	0.004 ± 0.001	0.005 ± 0.001	0.005 ± 0.002	**0.015**
O-Acetylcarnitine	HMDB00201	0.004 ± 0.003	0.010 ± 0.002	0.010 ± 0.006	0.011 ± 0.008	0.356
Ornithine	HMDB00214	0.014 ± 0.003	0.023 ± 0.005	0.020 ± 0.005	0.015 ± 0.004	**0.009**
Pantothenate	HMDB00210	0.001 ± 0.001	0.003 ± 0.002	0.002 ± 0.001	0.003 ± 0.001	0.190
Phenylalanine	HMDB00159	0.013 ± 0.006	0.034 ± 0.003	0.029 ± 0.007	0.026 ± 0.004	**<0.001**
Proline	HMDB00162	0.046 ± 0.023	0.127 ± 0.025	0.062 ± 0.019	0.058 ± 0.011	**<0.001**
Pyruvate	HMDB00243	0.022 ± 0.017	0.165 ± 0.022	0.102 ± 0.032	0.100 ± 0.022	**<0.001**
Sarcosine	HMDB00271	0.001 ± 0.001	0.001 ± 0.0005	0.001 ± 0.0003	0.001 ± 0.0001	**0.009**
Serine	HMDB00187	0.028 ± 0.019	0.124 ± 0.022	0.073 ± 0.020	0.060 ± 0.013	**<0.001**
sn-Glycero-3-phosphocholine	HMDB00086	0.001 ± 0.001	0.002 ± 0.002	0.001 ± 0.001	0.001 ± 0.001	**0.040**
Succinate	HMDB00254	0.016 ± 0.017	0.065 ± 0.025	0.039 ± 0.038	0.024 ± 0.013	0.051
Taurine	HMDB00251	0.128 ± 0.082	0.304 ± 0.088	0.170 ± 0.076	0.148 ± 0.078	**0.013**
Threonine	HMDB00167	0.052 ± 0.019	0.164 ± 0.034	0.119 ± 0.037	0.092 ± 0.047	**0.003**
Tyrosine	HMDB00158	0.020 ± 0.009	0.049 ± 0.014	0.035 ± 0.007	0.031 ± 0.007	**0.001**
Uracil	HMDB00300	0.009 ± 0.006	0.008 ± 0.004	0.006 ± 0.002	0.006 ± 0.002	0.165
Urea	HMDB00294	0.198 ± 0.066	0.639 ± 0.076	0.810 ± 0.984	0.929 ± 1.180	0.703
Valine	HMDB00883	0.039 ± 0.017	0.093 ± 0.014	0.062 ± 0.018	0.052 ± 0.015	**<0.001**

Legend: (A) control adult rat; (S) control ageing rat; (Wi) ageing tumour-bearing rat in intermediated stage; (Wa) ageing tumour-bearing rat in advanced stage. Data were expressed as mean ± (SD) standard deviation and analysed by one-way ANOVA (comparison among A, S, Wi and Wa). Bold *P* values represented a significant difference.
